# Effects of Bone Marrow Stromal Cell Transplantation through CSF on the Subacute and Chronic Spinal Cord Injury in Rats

**DOI:** 10.1371/journal.pone.0073494

**Published:** 2013-09-11

**Authors:** Norihiko Nakano, Yoshiyasu Nakai, Tae-Beom Seo, Tamami Homma, Yoshihiro Yamada, Masayoshi Ohta, Yoshihisa Suzuki, Toshio Nakatani, Masanori Fukushima, Miki Hayashibe, Chizuka Ide

**Affiliations:** 1 Institute of Regeneration and Rehabilitation, Aino University School of Nursing and Rehabilitation, Osaka, Japan; 2 Department of Occupational Therapy, Aino University School of Nursing and Rehabilitation, Osaka, Japan; 3 Department of Physical Therapy, Aino University School of Nursing and Rehabilitation, Osaka, Japan; 4 Department of Plastic and Reconstructive Surgery, Tazuke Medical Research Institute, Kitano Hospital, Osaka, Japan; 5 Emergency and Critical Care Center, Kansai Medical University, Osaka, Japan; 6 Translational Research Informatics Center, Foundation for Biomedical Research and Innovation, Kobe, Japan; Hertie Institute for Clinical Brain Research, University of Tuebingen., Germany

## Abstract

It has been demonstrated that the infusion of bone marrow stromal cells (BMSCs) through the cerebrospinal fluid (CSF) has beneficial effects on acute spinal cord injury (SCI) in rats. The present study examined whether BMSC infusion into the CSF is effective for subacute (1- and 2-week post-injury), and/or chronic (4-week post-injury) SCI in rats. The spinal cord was contused by dropping a weight at the thoracic 8-9 levels. BMSCs cultured from GFP-transgenic rats of the same strain were injected three times (once weekly) into the CSF through the fourth ventricle, beginning at 1, 2 and 4 weeks post-injury. At 4 weeks after initial injection, the average BBB score for locomotor assessment increased from 1.0–3.5 points before injection to 9.0-10.9 points in the BMSC-injection subgroups, while, in the PBS (vehicle)-injection subgroups, it increased only from 0.5–4.0 points before injection to 3.0-5.1 points. Numerous axons associated with Schwann cells extended longitudinally through the connective tissue matrices in the astrocyte-devoid lesion without being blocked at either the rostral or the caudal borders in the BMSC-injection subgroups. A small number of BMSCs were found to survive within the spinal cord lesion in SCI of the 1-week post-injury at 2 days of injection, but none at 7 days. No BMSCs were found in the spinal cord lesion at 2 days or at 7 days in the SCI of the 2-week and the 4-week post-injury groups. In an in vitro experiment, BMSC-injected CSF promoted the survival and the neurite extension of cultured neurons more effectively than did the PBS-injected CSF. These results indicate that BMSCs had beneficial effects on locomotor improvement as well as on axonal regeneration in both subacute and chronic SCI rats, and the results also suggest that BMSCs might function as neurotrophic sources via the CSF.

## Introduction

The treatment of spinal cord injury (SCI) has been extensively studied by transplantation of various kinds of cells, including bone marrow stromal cells [1,2,3,4,5,6,7,8], and cellular transplantation therapy using different kinds of cells has been extensively reviewed [9]. Of these cell types for transplantation, bone marrow stromal cells (BMSCs) are regarded as a promising candidate for clinical application, since they are seen to be highly effective in the repair of spinal cord injury in experimental animals. BMSCs are, in addition, readily and safely applicable as autologous transplants in clinical cases.

Our previous study demonstrated that BMSCs transplanted directly into the lesion contributed to locomotor improvement and axonal regeneration in rats with subacute (2 weeks post-injury) SCI [10]. The 2-week (2-w) post-injury (PI) is regarded as subacute or early chronic, and a 4-week PI is chronic. Houle and Tessler [11] reported that a 4-week PI is a chronic SCI based on the behavioral finding that locomotor recovery reached a plateau at ~ 4 weeks on BBB scores [12]. Other studies maintain that the acute period is less than 7 days, and the subacute is 1-2 weeks after contusion injury [13,14].

In the present study, we examined the effects of cell transplantation through the cerebrospinal fluid (CSF) on the subacute (1-w and 2-w PI) and chronic (4-w PI) SCI. We have already shown that the BMSC infusion into the CSF has beneficial effects on the acute SCI (immediately after injury) [3,15]. The study of BMSC-delivery via CSF by lumbar puncture also showed locomotor improvement in the acute SCI [16]. In the present study, GFP-transgenic BMSCs were injected three times (once weekly) into the fourth ventricle of rats with subacute and chronic SCI.

Cell transplantation through CSF is clinically preferable to surgical approaches since it involves no danger of inflicting secondary damages on remaining intact nerves of the host spinal cord. Based on the efficacy of BMSCs, and the safety of this cell injection method in rhesus monkeys, we proceeded to clinical application. BMSCs obtained from patients within 3 days following injury were administered by lumbar puncture [17,18].

On the other hand, patients waiting for treatment for a longer period are considered to have subacute or chronic SCI. It is therefore important to study whether BMSC injection through the CSF (via lumbar puncture) is as effective on patients with subacute and chronic SCI as it is expected to be on those with acute SCI [6,7,19,20,21].

In the present study, BMSCs were injected into the fourth ventricle 1-w, 2-w or 4-w PI in rats, and the locomotor improvements and the spinal cord repair were examined.

The results indicated that BMSCs, though having disappeared a short time after injection into the CSF, had beneficial effects on both locomotor improvement and tissue repair, including axonal regeneration, in both subacute and chronic SCI rats. The mechanisms for these beneficial effects are discussed below from the point of view of the function of BMSCs as neurotrophic sources via the CSF.

## Materials and Methods

### Surgery

Sprague-Dawley (SD) rats (6 weeks old, female) were used in the present study. The study was carried out with approval of the Animal Committee of Aino University, and all animal experiments were performed in accordance with the Guidelines for Animal Experiments of Aino University, laid down in compliance with the Japanese Regulations for Animal Welfare. The methods were basically the same as those described in the previous paper [10]. Briefly, rats were anesthetized by inhalation of isoflurane (1-2% Escain^®^, Mylan, Osaka, Japan) administered at a flow rate of 2 L/min. Laminectomy was performed at the Th8-9 vertebrates to expose the spinal cord. The exposed spinal cord was damaged by letting a metal weight (10g) drop free-fall from the height of 7.5 cm using an NYU impactor. Dropping a weight from a height of 5 cm is generally used for rat SCI. However, in our experiments, the BBB scores by this method tended to yield no difference between experiments and controls over a long survival time. Therefore, in the present study, we used the highest scale of the NYU device as we had done in our previous study [10].

Following injury, the layered muscles and skin were sutured to close the lesion. Immediately after surgery, animals were given 3 ml saline subcutaneously to avoid dehydration, and kept for a while on a warming pad until they awakened from the anesthesia. They were put individually into rat cages with absorbent chips. Small amounts of food were placed on the floor of the cage to enable the rats to access the food easily on the first postoperative day. Later, food and water were provided as usual from the top of the cage. As in the previous study, the animals were given an antibiotic (gentacin 3 mg/kg, Schering Plough, Osaka Japan) daily for 3 days to prevent infection. The bladders were manually expressed 3 times per day for 3 days. Rats were housed in an atmosphere of 55% humidity at a temperature of 23°C.

The number of rats used in the present study is shown in Table 1. Despite precautions, some rats died during the experiment. The physical condition of those that died varied: some appeared almost healthy until the day before death, while many others had difficulties in urination and defecation. Rats that gradually grew weak, losing body weight and vital forces, were sacrificed for humane reasons by intra-peritoneal injection of an overdose of anesthesia (pentobarbital, 50 mg/kg).

**Table 1:** Number of rats used.

category	injection	fixation periods after initial injection					
		2d	1w	2w	3w	4w	total
1W PI	BMSC	5 (Tr)	5 (Tr)	1 (IHC)		9 (IHC & HE: 6; TDA: 2; EM: 1)	30 (1)
		5 (CSF)	5 (CSF)				
	PBS	5 (CSF)	5 (CSF)			7 (IHC & HE: 6; EM: 1)	17 (2*)
2W PI	BMSC	5 (Tr)	5 (Tr)	2 (IHC)		9 (IHC & HE: 6; TDA: 2; EM: 1)	21 (1)
	PBS					6 (IHC & HE: 6)	6 (2*)
4W PI	BMSC	5 (Tr)	5 (Tr)	1 (IHC)		8 (IHC & HE: 6; TDA: 1; EM: 1)	19 (1)
	PBS					6 (IHC & HE: 6)	6 (3*)
total		25	25	4		45	99 (10)

Notes: BMSC: bone marrow stromal cell. PBS: phosphate buffered saline. IHC: immunohistochemistry. HE: hematoxylin and eosin staining. TDA: Texas Red-dextran amine labeling. EM: electron microscopy. Tr: Rats used for tracking the fate of transplanted BMSCs. CSF: Rats used for analysis of neurotrophic effects of CSF (cerebrospinal fluid). PI: post-injury.

The number following the abbreviations IHC & HE, TDA, and EM indicates the number of rats used for each method. Ten rats died during the procedure. This number is indicated in parentheses in the total-column of the Table.

(*) : A rat sacrificed for humane reasons is included.

### Cell culture

Four-week old GFP-transgenic SD rats were purchased from a local dealer (SLC, Osaka). From these, bone marrow tissues were obtained by perfusion through femurs and tibias with the culture medium and cultured in Dulbecco’s modified Eagle’s medium (DMEM) with 20% fetal calf serum (FCS). BMSCs adhered to the base of the culture flask, and proliferated to a density of ca. 5x10^6^ in one dish within 5-7 days of culture. The majority of the BMSCs were CD90 (+), CD29 (+), CD34 (-) and CD11b (-) by immunohistochemistry. Cells for transplantation were harvested by trypsin treatment followed by PBS-washing.

Neurons of the hippocampus were cultured for the bioassay of CSF effects on neurons. Neurons were cultured in the same way as described in the previous study [22]: the hippocampus was removed from the brain of newborn rats, and dissociated in Nerve Cell Dissociation Solution CP (DS Pharma Biomedical, Osaka, Japan). Dissociated cells were seeded on poly-L-lysine (PLL)-coated 48-well microplates, and cultured in Neurobasal Medium containing B27 supplement (Invitrogen, Carlsbad, Ca. USA), 2mM L-glutamine, penicillin, and streptomycin (MB/B27 medium). After 12 h incubation, neurons were re-fed with the DMEM containing the CSF to be analyzed, and were further incubated for 24 hours. The concentration of the CSF was 5% in 400 µl of DMEM. Survival of neurons and extension of neurites were estimated at 24 hours of incubation.

### Cell transplantation

**Figure 1:**
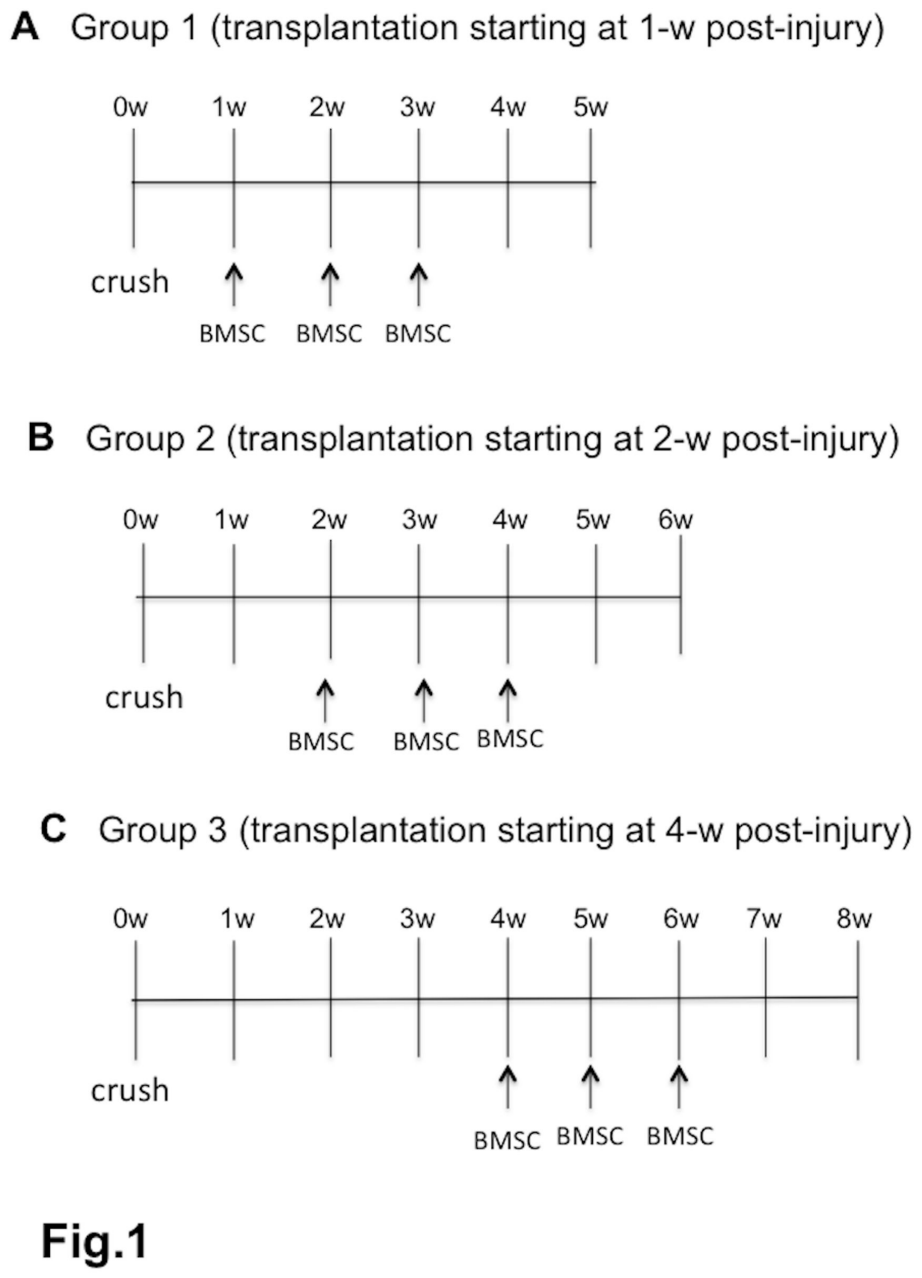
Experimental designs. The figure shows the three experimental groups of cell injection. BMSCs in PBS were injected into the CSF three times (once weekly) into the 4th ventricle 1, 2, or 4 weeks after contusion injury to the spinal cord. For the control, the vehicle (PBS) without BMSCs was injected in the same manner. Cell- or vehicle-injection started at 1 week post-injury (PI) in Group 1 (A), at 2 weeks PI in Group 2 (B), and at 4 weeks PI in Group 3 (C). Most rats were fixed at 4 weeks following the initial injection. A few rats were fixed 2 weeks after the initial injection.

Cell transplantation was performed 1-w, 2-w, and 4-w PI (Figure 1). The rats were anesthetized by inhalation of isoflurane, and a pore (0.5 mm diameter) was made in the skull at 3.5 mm caudal to the lambda suture in the midline. BMSC suspension at a density of 5x10^6^ in 50 µl phosphate-buffered saline (PBS) was injected into the fourth ventricle of rats using an insulin syringe with a 29-gauge needle. On the stereotactic device (model SR-6N, Narishige, Japan), the needle was inserted vertically to a depth of 6 mm from the cerebellar surface. The injection time was 2 min, and the needle was retained for 2 min after injection (cell transplantation group). After injection, the skin was sutured. The cell injection was performed three times (once weekly) in the same manner (Figure 1). For the control, PBS without BMSCs was injected in the same manner as above. In the preliminary experiments, there was no difference in BBB scores and spinal cord repair in two groups: the PBS-injection and non-treatment groups. Therefore, only PBS injection was used for the control in the present study.

For tracking the fate of transplanted BMSCs, the BMSC suspension was transplanted once in each of the 1-w, 2-w and 4-w PI groups in the same manner as above. Rats were fixed 2 and 7 days after transplantation. Ten rats each were used from the 1-w, 2-w and 4-w PI groups. Of each of these 10 rats, 5 were fixed at the time point of 2 days and 5 at 7 days after the transplantation in each group (Table 1).

### CSF sampling and CSF effects on neurons in vitro

Based on the hypothesis that transplanted BMSCs might release into the CSF certain molecules effective in promoting tissue repair and axonal regeneration, a preliminary experiment was performed to examine the effects on cultured neurons of the CSF derived from BMSC-transplanted and PBS-injected rats. BMSCs were transplanted once to 10 rats, and PBS was injected once into 10 rats in the 1-w PI group. For sampling the CSF, after rats were anesthetized by inhalation of isoflurane, a 50 µl of CSF was harvested from the cisterna magna with a tuberculin syringe 2 and 7 days after BMSC transplantation or PBS injection. From the 10 rats of each subgroup, 5 rats were used at the time points of 2 days and 7 days after transplantation (Table 1).

For bioassay of CSF effects, hippocampus neurons were cultured, and the sampled CSF was mixed with the culture medium at a concentration of 5%, as described above. For assessing the density of the neurons and the neurite length, neurons from 5 cultures in the medium containing CSF derived from BMSC-transplanted and PBS-injected subgroups were photomicrographed using a 10X objective. Three representative micrographs were selected for each of the 5 cultures. The number of neurons and the length of neurites were measured in the 15 micrographs taken at each of the two time points in the BMSC-transplanted subgroup and 15 taken at each of the same two time points in the PBS-injected subgroup, using the Image Filing System (Flovel, Tokyo) equipped on the microscope (0lympus CKX41, Tokyo). The density of neurons was expressed as values per area of 0.25 mm^2^, and the neurite length was calculated by dividing the total neurite length by the number of neurons.

### Locomotor assessment

The locomotor behaviors were assessed on the BBB (Basso-Beattie-Bresnahan) scale [12] in an open area (ca. 1.0 m^2^). Rats were observed for 5 min every week after cell transplantation or PBS injection until the end of the experiment. Locomotion was recorded by digital video camera at each time point, and analyzed by two researchers who were not aware of the experiment. The scale ranges from 1 point (no observable hind limb movement) to 21 (consistent plantar stepping and coordinated gait, consistent toe clearance, predominant paw position parallel throughout stance, consistent trunk stability, and tail consistently up). Movements of three joints (hip, knee, and foot) of the hind limb, plantar placement of hind foot, body weight support, and forelimb and hind limb coordination are critical checking points for the assessment of locomotor recovery. BBB scores of 9-10 indicate that rats can support their body weight in walking with plantar placement of hind feet.

### Histology and immunohistochemistry

For histological and immunohistochemical experiments, a total of 40 rats were sacrificed as follows: 6 rats each for the cell injection and the control subgroups were used at 4 weeks after the initial injection in each (1-w, 2-w, and 4-w PI) group. In addition, 4 rats were fixed for immunohistochemistry at 2 weeks after initial injection (Table 1).

For fixation, rats were initially anesthetized by an inhalation of isoflurane as above, and then given an intra-peritoneal injection of pentobarbital (50 mg/kg) for deep anesthesia. The mouthpiece for inhalation was removed from the rat, and the thorax was opened to expose the heart. A cannula was inserted into the aorta through the left ventricle, through which rats were perfused by a roller pump with 50 ml PBS, followed by 200 ml of a fixative containing 2% paraformaldehyde in 0.1M phosphate buffer (pH 7.4). Specimens were excised, stored in the same fixative for 1-2 days, immersed for 1 day each in 10, 20 and 30% sucrose solution (0.1M phosphate buffer), embedded in OCT compound, and cut coronally or horizontally into 7 µm-thick frozen sections with a cryostat.

For histology, horizontal sections were stained with hematoxylin and eosin (HE). The tissue repair and cavity formation in the lesion were examined in each group.

For immunohistochemistry, after washing and blocking, sections were incubated overnight with a solution containing primary antibodies as follows: anti-glial fibrillary acidic protein (GFAP) monoclonal antibody (1:300; Sigma) for astrocytes, anti-neurofilament 200 kDa rabbit polyclonal antibody (1:100; Chemicon) for axons, anti-GST-πrabbit polyclonal antibody (1:300; MBL) for oligodendrocytes, anti-5HT rabbit polyclonal antibody (1:2000; Sigma) for raphespinal fibers, anti-TH mouse monoclonal antibody (1:150; Chemicon) for coerulospinal fibers, anti-CGRP rabbit polyclonal antibody (1:2000; Sigma) for primary sensory fibers, and anti-mouse integrinαM (OX42, 1:500; Santa Cruz) for macrophages. After being washed, the sections were incubated overnight with secondary antibodies as follows: FITC- or Cy-3-labeled anti-mouse IgG antibody (1:1000; Amersham Bioscience) for astrocytes, coerulospinal fibers, and macrophages, and Cy-3-labeled anti-rabbit IgG antibody (1:1000; Amersham Biosciences) for oligodendrocytes, axons, raphespinal fibers, and primary sensory fibers. Sections were mounted and examined by a fluorescence microscope (Axio Imager MI; Carl Zeiss).

For tracking the fate of transplanted BMSCs, we examined the survival of GFP-positive BMSCs within the spinal cord lesion. For detection of GFP-positive BMSCs, 30 rats were sacrificed: 5 rats at each of the time points of 2 days and 7 days after cell transplantation were used in the 1-w, 2-w, and 4-w PI groups (Table 1). Rats were fixed, and specimens of spinal cord were prepared and processed in the same manner as in the case of immunohistochemistry (previous paragraph). Sections were observed in a fluorescence microscope after immunohistochemical staining for GFAP to display astrocyte distributions on a background.

### Demonstration of corticospinal axons

One week before sacrifice, rats were injected with Texas Red-labeled dextran amine (TDA, Molecular Probes, Eugene, Oregon, USA) for labeling the corticospinal axons within the lesion. One or two rats in each cell transplantation subgroup were used for TDA labeling at 4 weeks after initial injection (Table 1). Under anesthesia, rats were placed on the stereotaxic device, and holes were made with a drill in the skull at 4 points: 1.0 and 2.0 mm caudal to the bregma, and 2.5 mm lateral to the midline on both sides. An insulin syringe was used for the injection. The needle was inserted 1.5 mm deep from the brain surface, and the 10% TDA solution (1-2 µl) was injected for a duration of 3 min. One week after injection, rats were anesthetized by inhalation of isoflurane, followed by an intra-peritoneal injection of pentobarbital, and transcardially perfused in the same manner as above. The segment of spinal cord lesion, including adjacent areas, was excised and stored in the same fixative for one day. After immersion in sucrose solution, specimens were embedded in the OCT compound. Cryostat sections were observed in a fluorescence microscope.

### Electron microscopy

One rat in each cell transplantation subgroup was fixed for electron microscopy at 4 weeks after the initial injection. Also at 4 weeks after initial injection, one control rat in the 1-w PI group was fixed (Table 1). Rats were anesthetized by inhalation of isoflurane, given an intra-peritoneal injection of pentobarbital in the same manner as above, and transcardially perfused with 50 ml PBS, followed by perfusion of 100 ml fixative containing 1% glutaraldehyde and 1% paraformaldehyde in 0.1 M phosphate buffer, pH 7.4. The spinal cord, including the injured site, was stored in the same fixative for 1 day, and transversely cut into several tissue slices. These tissue slices were then post-fixed in 1% osmium tetroxide in 0.1 M phosphate buffer, dehydrated, and embedded in Epon 812. Thin Epon sections were cut with a diamond knife on an ultramicrotome (Ultracut UCT, Leica), stained with uranyl acetate and lead citrate, and observed by electron microscopy (H 7650, Hitachi, Tokyo). Semi-thin Epon sections were stained with toluidine blue for observation by light microcopy.

### Fluorescence density of astrocytes

Rats fixed 4 weeks after the initial cell- or vehicle-injection were used. Astrocytes were immunostained for GFAP as described above. Since occasionally no astrocytes could be found at the epicenter, coronal (transverse) sections of spinal cord at 1-mm rostral and at 1-mm caudal to the epicenter were used for measuring the density of fluorescence of astrocytes. From among the BMSC-transplanted rats, 3 rats each were used from the 1-w, 2-w and 4-w PI groups, and from among the PBS-injected (control) rats, 3 were used from the 4-w PI group.

Fluorescence density was measured using ImageJ at 4 points in the right half and 4 points in the left half of the transverse section: 2 at the near-midline on the border of the lesion, 2 on the outer surface of the spinal cord; and 2 at lateral site on the border of the lesion, 2 on the outer surface of the spinal cord.

Since the fluorescence density differed subtly from section to section, it is reasonable to compare fluorescence densities within the same section. In the present study, the fluorescence densities were measured at 4 points at the outer surface as described above, and their average values were used as the reference for comparisons within each group. The fluorescence density at the border of the lesion was expressed in percentage of the average value on the outer surface.

### Cavity volume

Rats were fixed 4 weeks after the initial cell or vehicle injection. Three rats each for BMSC-transplanted and PBS-injected subgroups in the 1-w, 2-w, and 4-w PI groups were used. The spinal cord segment was cut horizontally into 7 µm-thick frozen sections on cryostat, and stained with HE. The areas of the cavities were measured in every fifth section (at 35-μm intervals) using the Image Filing System (Flovel, Tokyo) equipped on a microscope (Olympus, CKX41, Tokyo). The cavity volume was calculated by multiplying the average area of the cavities by the total depth of the sections examined. The areas containing only free cells or scanty cell dispersion were regarded functionally as cavities. The relative volume (%) of the cavity was calculated by dividing the values of cavity volume as measured above by those of the whole spinal cord volume at the corresponding level.

### Counting of axons in immunohistochemical preparations

Axons were counted in the astrocyte-devoid area at the epicenter of transverse sections double-stained for astrocytes and neurofilaments. Individual cross-sectioned axons were identified as strong fluorescent points, and obliquely cut axons were identified as weak fluorescent strands. When several axons assembled, only identifiable strong fluorescent points or strands were counted. Three spinal cords each were used from BMSC-transplanted rats and control rats in the 4-w PI group.

### Statistics

The BBB scores were analyzed by repeated measures ANOVA, and post-hoc analysis was performed by Bonferroni’s test to assess the significance of differences at each time point (P<0.05 or P<0.01).

For cavity formation, CSF effects on neurons in vitro, and axon density within the lesion, the mean values were analyzed using Student’s t-test for the difference between the cell transplantation and the control groups. For astrocytic gliosis, mean values of fluorescence density for GFAP were analyzed using Student’s t-test for the difference between the border of lesion and the outer surface of the spinal cord in both the BMSC-transplanted and the PBS-injected subgroups.

## Results

### Cavity formation

**Figure 2:**
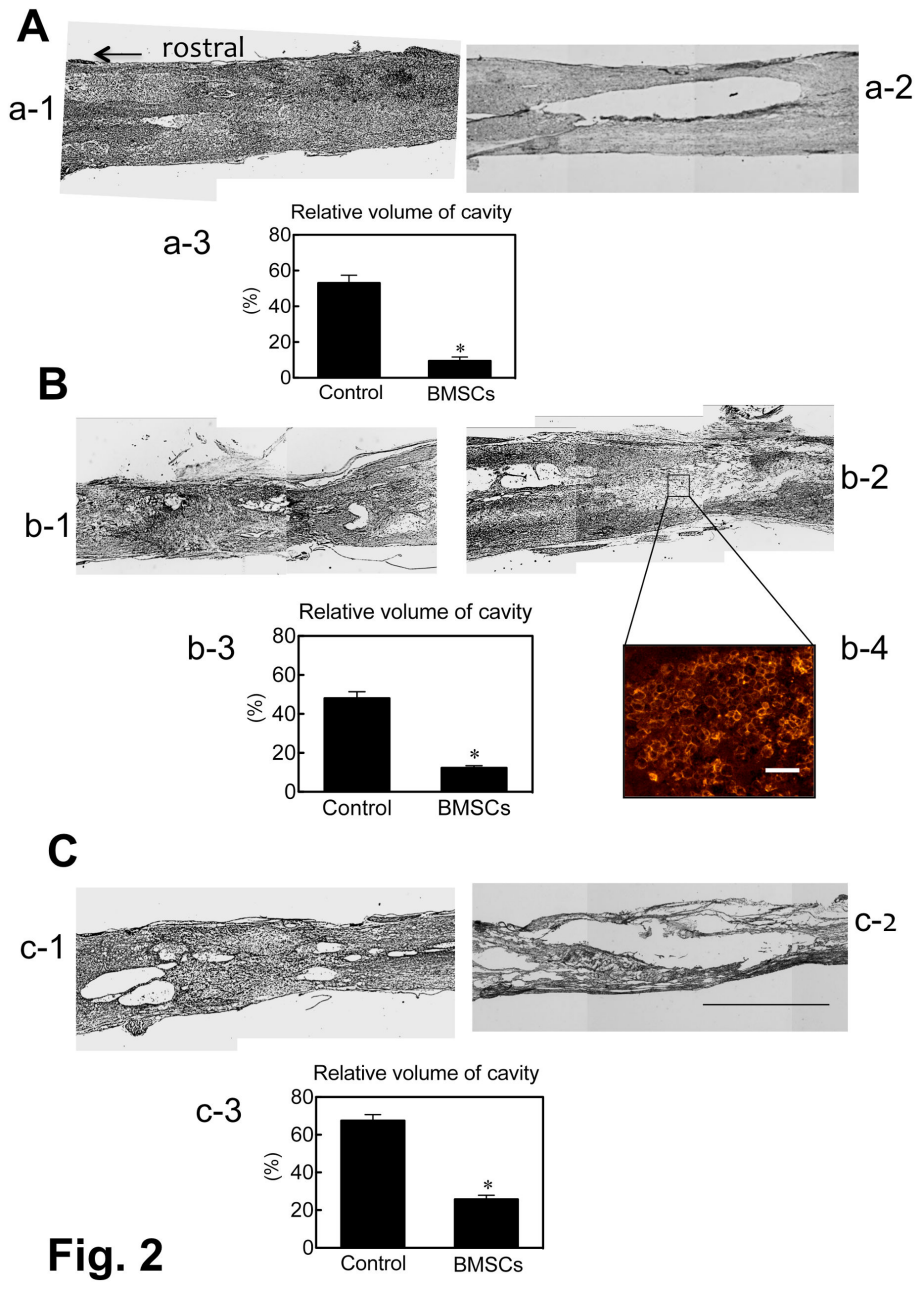
Differences in cavity formation between the BMSC-transplanted and the PBS-injected (control) rats. The pictures show the representative horizontal sections of the spinal cord from rats of 1-w, 2-w and 4-w PI. HE-staining. (A) Spinal cord sections of BMSC-transplanted (a-1) and PBS-injected (a-2) rats at 1-w PI. The cavity volume (a-3) relative to the whole spinal cord volume was 55.5 ± 5.3% (mean ± SEM) in the control, and 10.4 ± 3.5% in the BMSC-transplanted rats (* p < 0.05). R: rostral direction for all horizontal sections. (B) Spinal cord sections of BMSC-transplanted (b-1) and PBS-injected (b-2) rats at 2-w PI. The cavity volume (b-3) relative to the whole spinal cord volume was 50.2 ± 5.5% in the control, and 13.5 ± 2.0% in the BMSC-transplanted rats (* p < 0.05). b-4: Immunohistochemistry for macrophages from the spinal cord lesion of the section corresponding to the site shown in b-2. Macrophages are immunostained for OX42 on the cell surface. Scale: 100 µm. (C) Spinal cord sections of BMSC-transplanted (c-1) and PBS-injected (c-2) rats at 4-w PI. The cavity volume (c-3) relative to the whole spinal cord volume was 70.5 ± 5.0 in the control, and 31.8 ± 3.7% in the BMSC-transplanted rats (* p < 0.05). Scale: 1 mm for all HE-stained sections.

**Figure 3:**
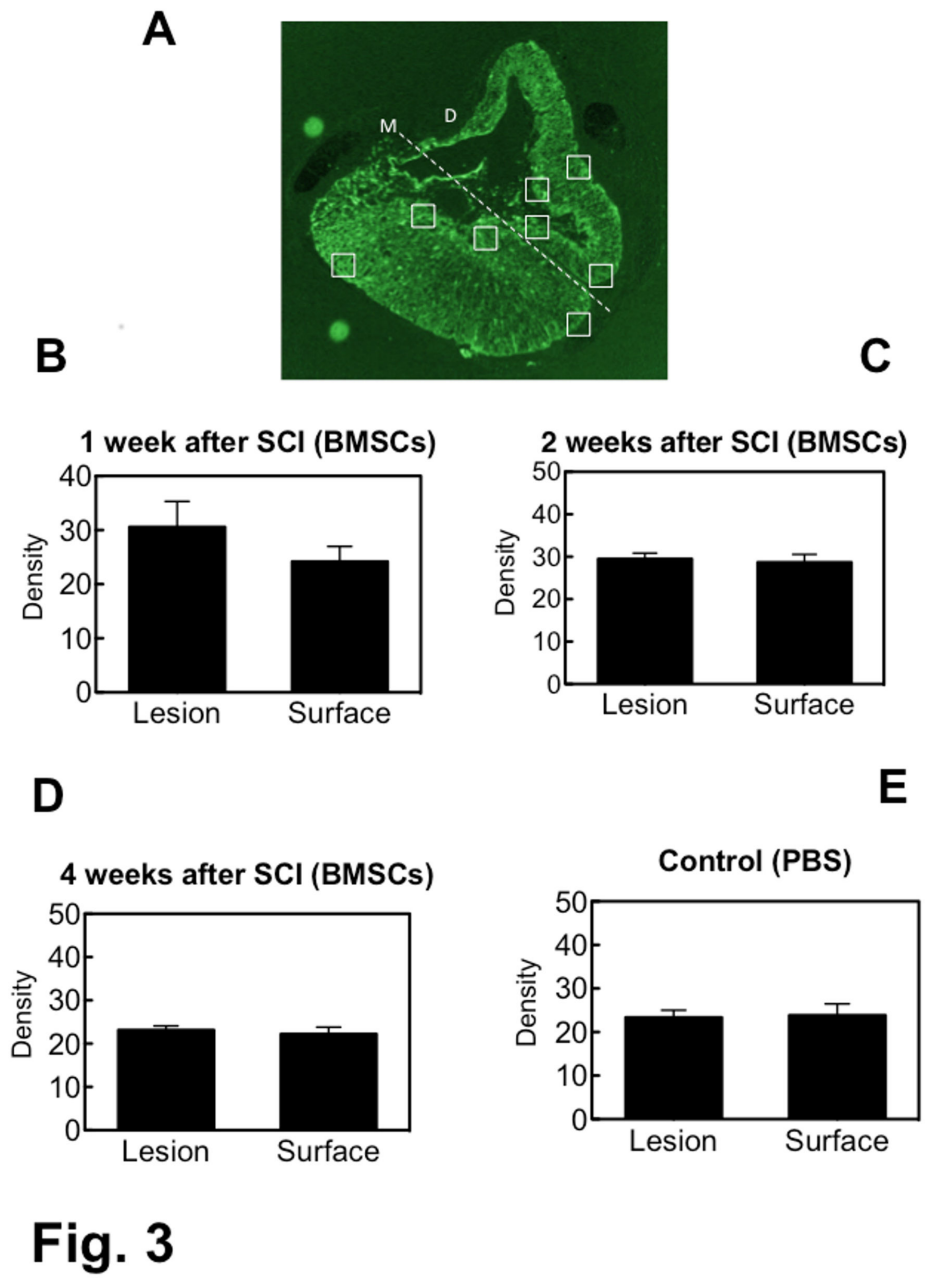
Fluorescence density of FITC-labeled astrocytes. An example of a transverse section of spinal cord, in which the fluorescence density was measured, is presented in (A). This section is the same as shown in Figure 6A. Fluorescence density was measured using ImageJ at 4 points (the rectangles) in the right half and 4 points in the left half of the transverse section: 2 at the near-midline on the border of the lesion and 2 on the outer surface of the spinal cord; 2 at lateral site on the border of the lesion and 2 on the outer surface of the spinal cord. M: midline, D: dorsal side of the spinal cord. The graphs show the fluorescence density of astrocytes in the 1-w (B), 2-w (C) and 4-w PI (D) groups of BMSC-transplanted subgroups, and in the 4-w PI group of PBS-injected (control) subgroup (E). There is no significant difference in fluorescence density between the border of the lesion and the outer surface of the spinal cord in any of the groups.

**Figure 4:**
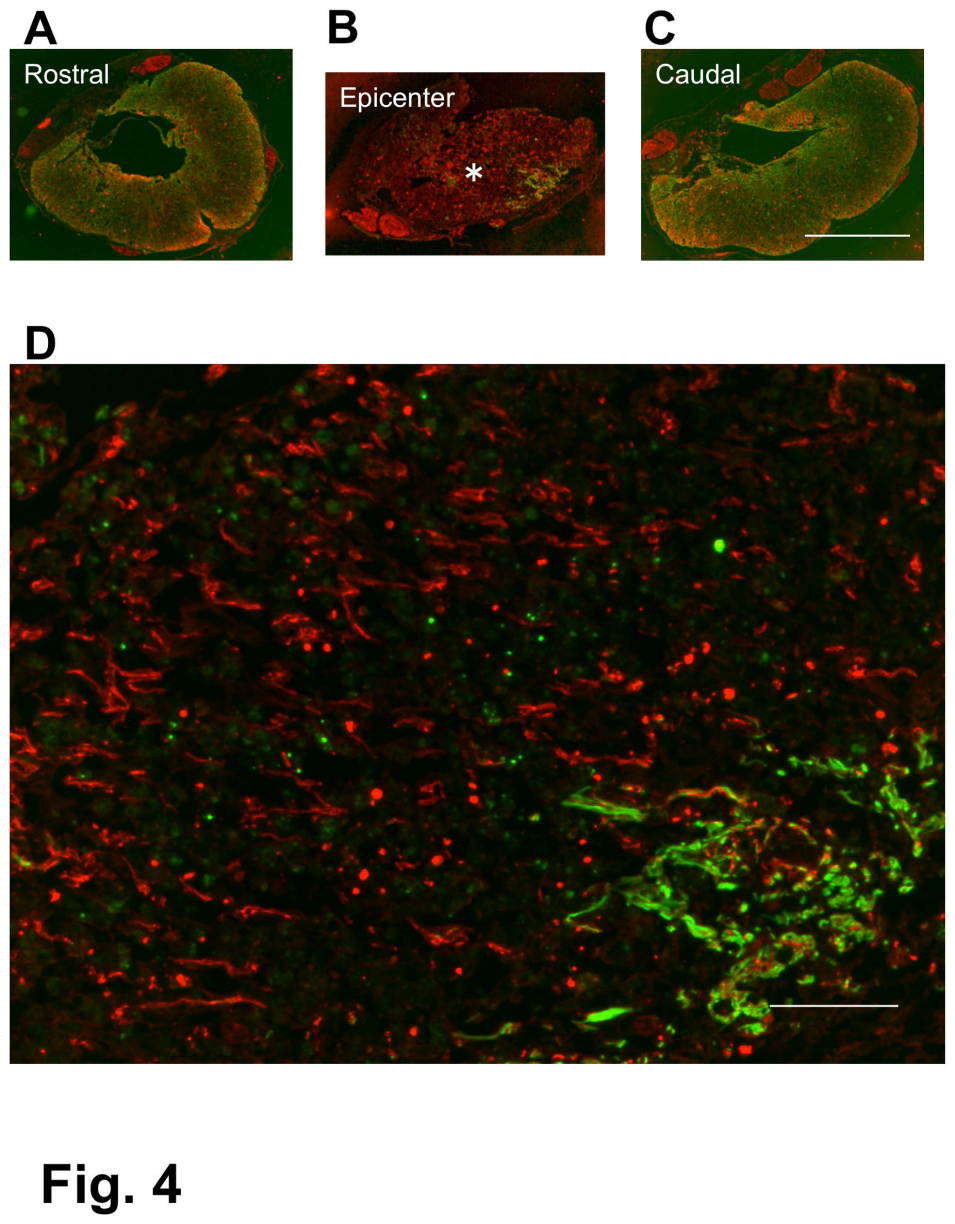
Spinal cord of a BMSC-transplanted rat. 1-w PI. Immunostaining for axons (red) and astrocytes (green). Transverse section. This spinal cord was obtained from the rat that had received BMSC transplantation starting at 1 week after contusion injury and was fixed 4 weeks following the initial cell injection. This rat showed BBB improvement from 1 point before cell injection to 11 at fixation. The panels (A), (B), and (C) show cross-sections of the spinal cord 1-mm rostral to the epicenter, epicenter of the lesion, and 1-mm caudal to the epicenter, respectively. The spinal cord becomes narrow at the epicenter (B), where almost no astrocytes are seen, while numerous axons run through the central area of the lesion. A part of the epicenter (*) in (B) is enlarged in (D), showing numerous axons at the center, and a small number of remaining astrocytes at the periphery of the spinal cord. Scale: 1 mm for (A), (B), and (C); 125 µm for (D).

The lesion of the spinal cord was occupied by varying amounts of parenchymal tissue in the BMSC-transplanted rats. The average cavity volume was 10.4 ± 3.5%, 13.5 ± 2.0%, and 31.8 ± 3.7% (means ± SEM) in 1-w, 2-w and 4-w PI groups, respectively, of the BMSC-transplanted subgroups (Figure 2). In contrast, a large space remained empty or was occupied by loosely dispersed cellular components in the control. Since these loosely dispersed cellular components, consisting mainly of macrophages (Figure 2, b-4), appeared to have no functional relationship with the surrounding host spinal cord tissue, the space occupied by such cellular components was regarded in the present study as part of the cavity. The cavity formation was clear in the control. The average cavity volume of the PBS-injected subgroups was 55.5 ± 5.3%, 50.2 ± 5.5%, and 70.5 ± 5.0% in 1-w, 2-w and 4-w PI groups, respectively (Figure 2). There are significance differences (p < 0.05) in cavity volume between the BMSC-transplanted and the PBS-injected subgroups in all three groups.

In most cases, numerous macrophages appeared following spinal cord injury in the lesion in an early period of injury. In the cases in which no tissue repair occurred, macrophages remained for a long time within the lesion. Cavities were formed therein after macrophages had disappeared from the lesion. In the case in which a tissue repair occurred, varying amounts of parenchymal tissues, including regenerating axons, appeared within the spinal cord lesion. A small number of macrophages were dispersed within the lesion, even when the lesion appeared to be occupied by regenerating parenchymal tissues.

### Astrocytic gliosis

Astrocytic gliosis was evaluated by measuring the fluorescence density for GFAP at the border of the lesion and at the outer surface of the spinal cord (Figure 3). The fluorescence density at the outer surface of the spinal cord was used as the reference for comparison. Since astrocytes were often lacking at the epicenter of the lesion, the fluorescence density of astrocytes was measured at the rostral and caudal parts of the lesion. The density rates at the border of the lesion were 126.8 ± 10.27%, 105.5 ± 7.29% and 106.9 ± 6.27% relative to the density at the outer surface, in the 1-w, 2-w and 4-w PI groups of the BMSC-transplanted subgroup, respectively. In the PBS-injected (control) subgroup, the density rate at the border of the lesion was 99.5 ± 5.53% relative to the density at the outer surface. There was no significant difference in fluorescence density between the border of the lesion and the outer surface of the spinal cord in any of the groups. This meant that there was no distinct astrocytic gliosis at the border of the lesion in the BMSC-transplanted and the PBS-injected rats.

### Axonal outgrowth

**Figure 5:**
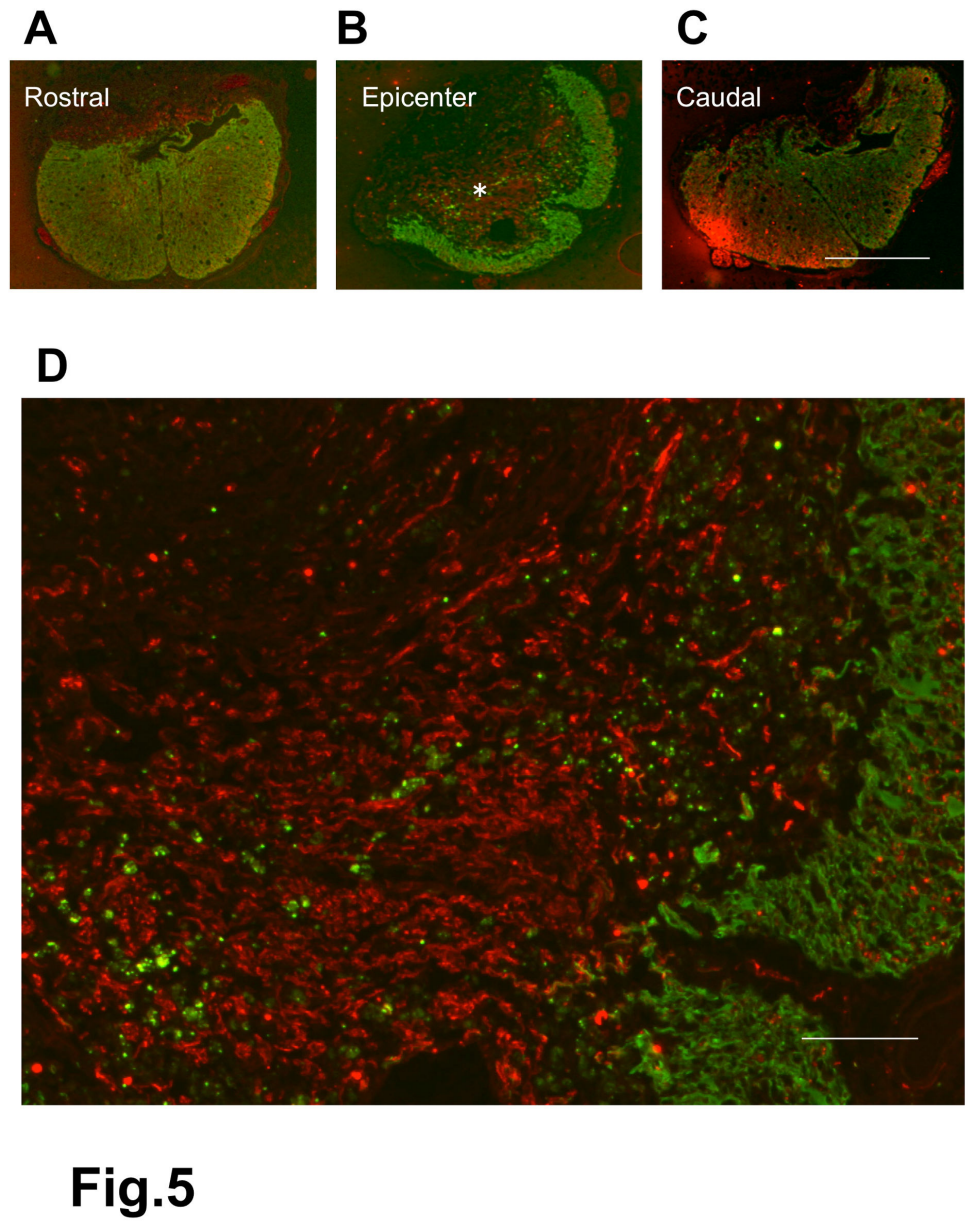
Spinal cord of a BMSC-transplanted rat. 2-w PI. Immunostaining for axons (red) and astrocytes (green). Transverse section. This spinal cord was obtained from the rat that had received BMSC injections starting at 2 weeks after contusion injury and was fixed 4 weeks following the initial cell injection. This rat showed BBB improvement from 3 points before cell injection to 9 at fixation. The panels (A), (B), and (C) show the cross sections of the spinal cord 1-mm rostral to the epicenter, epicenter of the lesion, and 1-mm caudal to the epicenter, respectively. The spinal cord at the epicenter (B) has, on the right margin of the spinal cord, a part of the white matter (shown in green) remaining after injury, with numerous axons extending in the central area of the lesion. A part of the epicenter (*) in (B) is enlarged in (D), showing numerous axons and a small number of astrocytes remaining on the margin of the spinal cord. Scale: 1 mm for (A), (B), and (C); 125 µm for (D).

**Figure 6:**
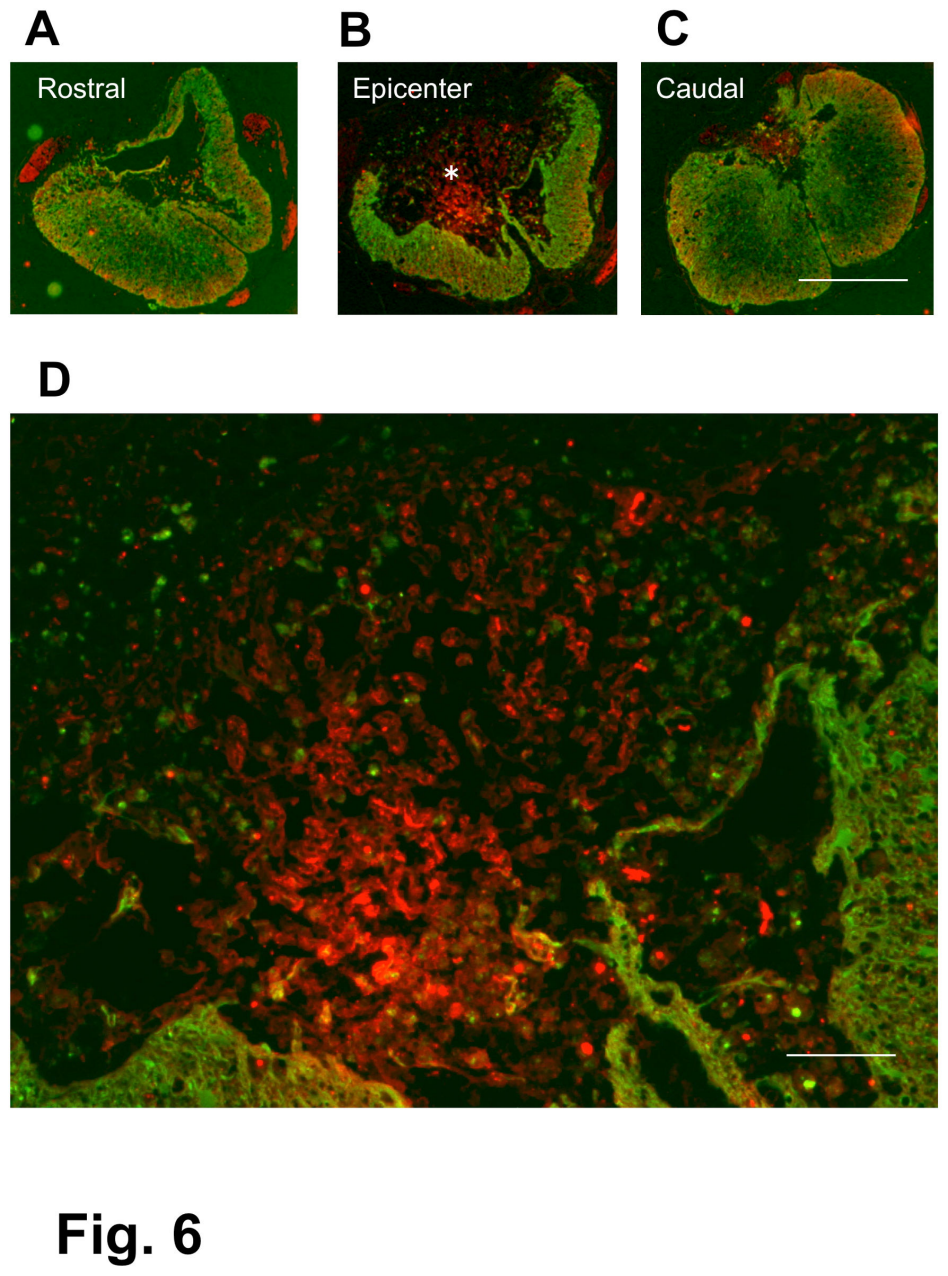
Spinal cord of a BMSC-transplanted rat. 4-w PI. Immunostaining for axons (red) and astrocytes (green). Transverse section. This spinal cord was obtained from the rat that had received BMSC injections starting at 4 weeks after contusion injury and was fixed 4 weeks following the initial cell injection. This rat showed BBB improvement from 4 points before cell injection to 10 at fixation. The panels (A), (B), and (C) show cross-sections of the spinal cord 1-mm rostral to the epicenter, epicenter of the lesion, and 1-mm caudal to the epicenter, respectively. The spinal cord at the epicenter (B) shows a part of the white matter remaining at the ventral margin of the spinal cord, with numerous axons extending in the central area of the lesion. A part of the epicenter (*) in (B) is enlarged in (D), showing numerous axons and a small number of astrocytes remaining at the periphery of the spinal cord. Scale: 1 mm for (A), (B), and (C); 125 µm for (D).

**Figure 7:**
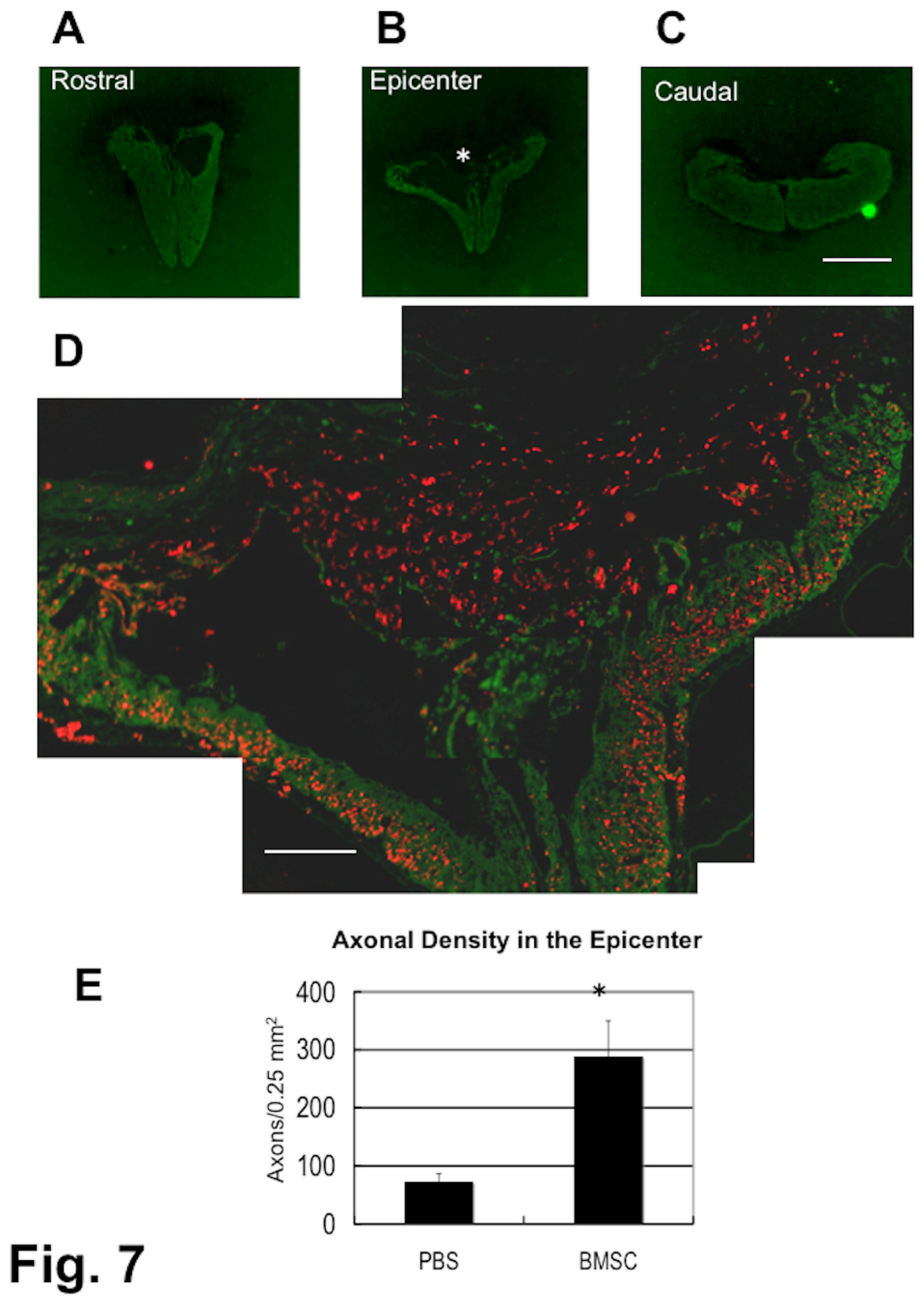
Spinal cord of PBS-injected (control) rat. 4-w PI. Immunostaining for axons (red) and astrocytes (green). Transverse section. This spinal cord was obtained from the rat that had received PBS injections starting at 4 weeks after contusion injury, and was fixed 4 weeks following the initial injection. The BBB score of this rat was 6 points before PBS injection and 8 points at 4 weeks after the initial injection. The panels (A), (B), and (C) show cross-sections of the spinal cord 1-mm rostral to the epicenter, epicenter of the lesion, and 1-mm caudal to the epicenter, respectively. A part of the white matter remains at the ventral zone of the epicenter spinal cord (B). A part of the epicenter (*) in (B) that was double-immunostained for axons and astrocytes is enlarged in (D). Very few axons are seen in the central area of the lesion, with a thin layer of white matter containing some axons at the ventral region of the spinal cord. Panel (E) shows the difference between BMSC-transplanted and PBS-injected (control) rats in axon density in the astrocyte-devoid area (* p < 0.01). The number of axons was counted in the astrocyte-devoid area, and divided by the size of the astrocyte-devoid area. Three spinal cords each from the 1-w, 2-w and 4-w PI groups of BMSC-transplanted subgroup and 3 from the 4-w PI group of PBS-injected subgroup were used for this quantification. Scale: 1 mm for (A), (B), and (C); 125 µm for (D).

Numerous axons were found within the epicenter of the lesion in the transverse section of the spinal cord in all three groups (Figures 4, 5, 6). These axon bundles were longitudinally oriented through the central part of the lesion. It should be noted that there was no immunostaining for astrocytes or oligodendrocytes in the epicenter of the lesion. Only a small amount of host spinal cord tissue immunostainable for GFAP remained at the periphery of the injured spinal cord. Oligodendrocytes were found with astrocytes, and, therefore, the data concerning the localization of oligodendrocytes are not shown in the present study. It is remarkable that numerous axons extended through such astrocyte-devoid areas. These findings were the same in the three groups. Even in a chronic spinal cord injury, a large number of axons were observed within the epicenter of the lesion (Figure 6). The control rats showed only a few axons within the epicenter of the spinal cord lesion in the 4-w PI group (Figure 7A–D). The densities of axons were 291.6 ± 64.7, and 65.5 ± 15.3/0.25mm^2^ in the BMSC-transplanted and in the PBS-injected subgroups（p < 0.01), respectively (Figure 7E). The cell-transplanted rats exhibited a significantly higher axon density than the PBS-injected rats.

**Figure 8:**
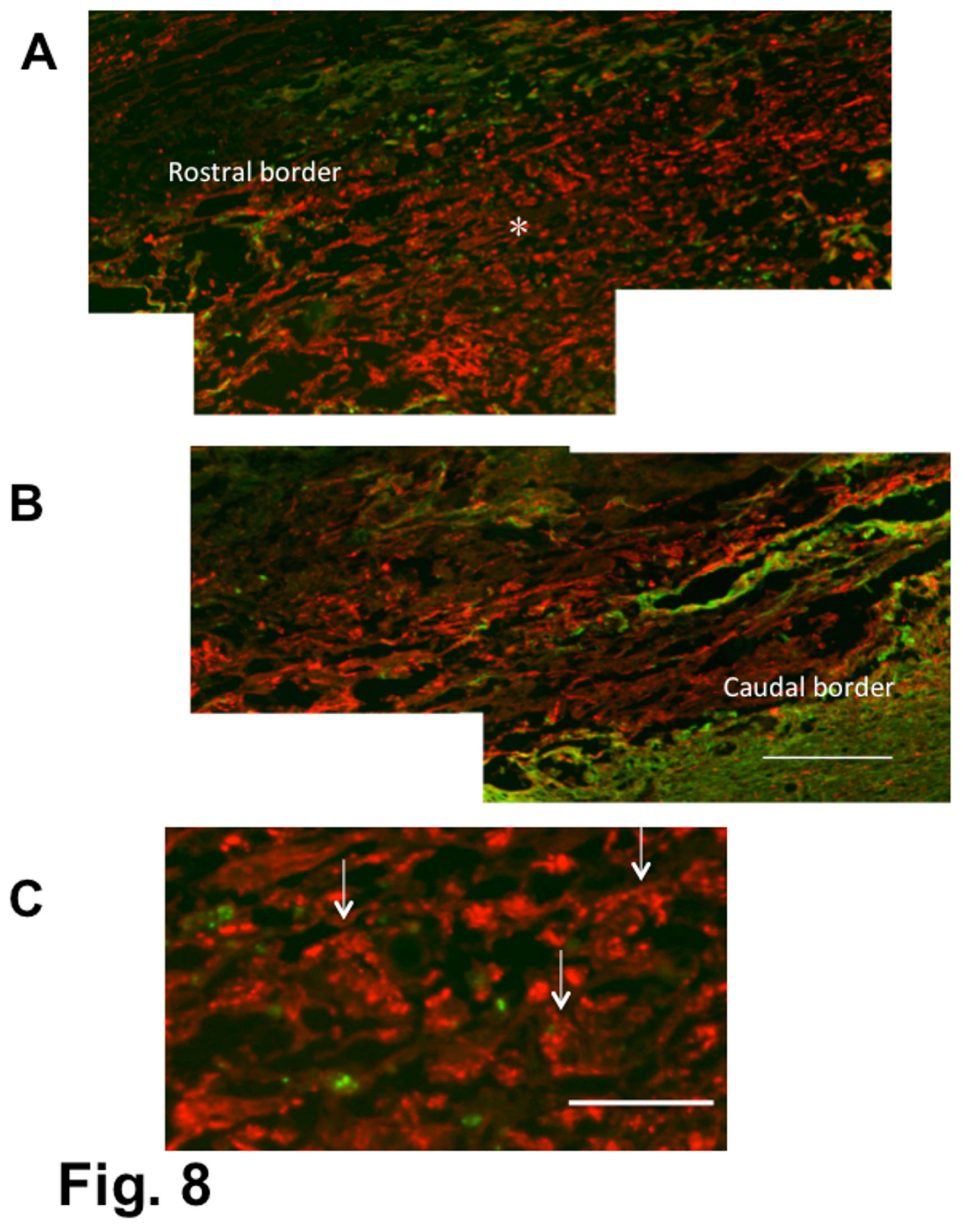
Spinal cord of a BMSC-transplanted rat. 4-w PI. Immunostaining for axons (red) and astrocytes (green). Longitudinal (horizontal) section. The rat had received BMSC transplantations starting at 4 weeks after contusion injury and was fixed 4 weeks following the initial cell injection. The BBB score of the rat was 4 points before cell injection and 12 points at fixation. Panels (A) and (B) show the rostral and caudal borders of the lesion, respectively. Some astrocytes are found at the borders. Panel (C) shows an enlargement of a part (*) of the epicenter in (A). There are numerous axons traversing through the astrocyte-devoid area. It appears that the axons traversing the lesion are not blocked from extending at the rostral or caudal border. Axons appear in most cases grouped in small fascicles (arrows). Scale: 200 µm for (A) and (B), and 50 µm for (C).

In the longitudinal section, there was no finding suggesting a blocked axonal extension at the rostral or caudal borders (Figure 8A, B). It appeared that axons traversing the lesion extended further through the rostral or caudal borders. Regenerating axons were in most cases grouped in small fascicles (Figure 8C).

**Figure 9:**
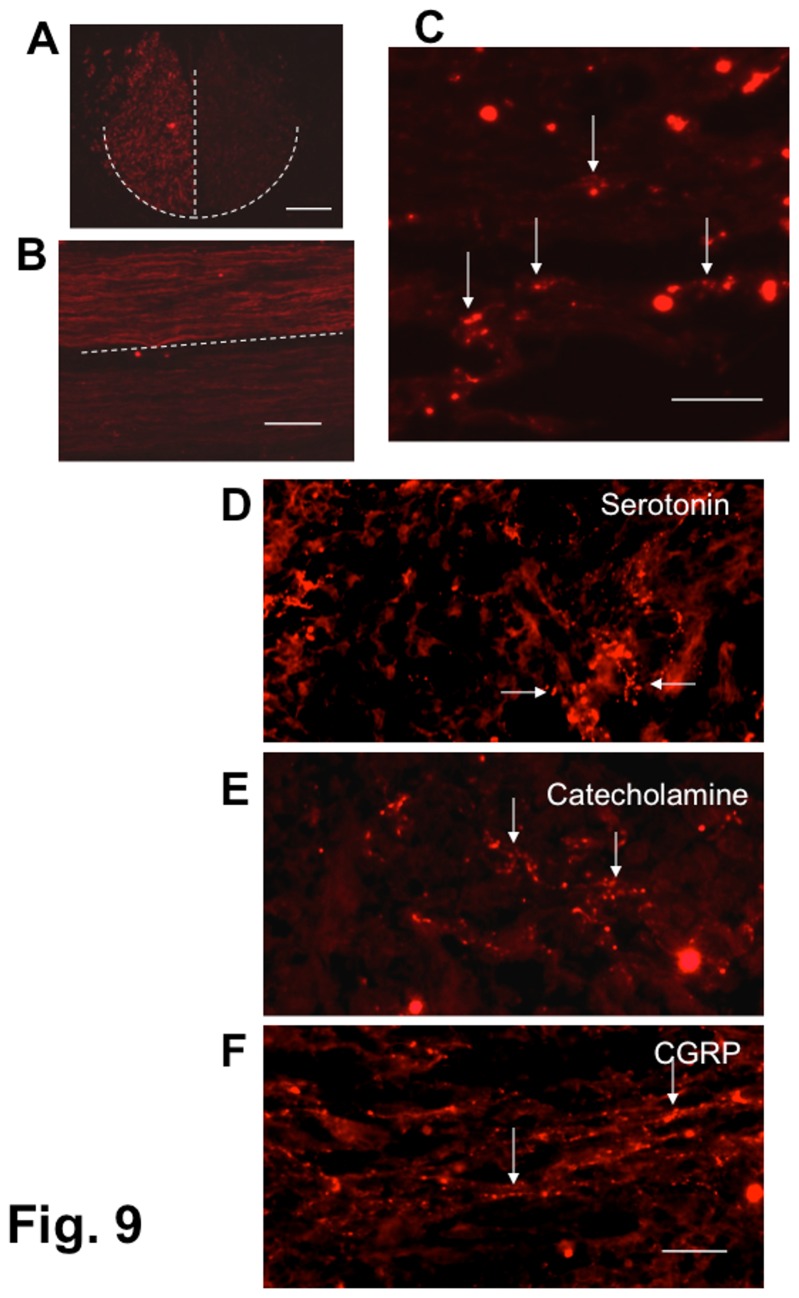
Detection of corticospinal, serotonergic, monoaminergic and CGRP-containing fibers. Panels (A), (B), and (C): Texas red-dextran amine labeling of corticospinal fibers. Texas red-dextran amine was injected into the motor area of the cerebral cortex on both sides of the rat 1 week before fixation. The rat had received BMSC transplantations starting at 4 weeks after contusion injury (4-w PI group). The rat showed a BBB improvement from 4 points before cell injection to 9 at 4 weeks following the initial cell injection. (A) Cross-section of the upper thoracic spinal cord (intact region) of a SCI rat. Texas Red-labeled fibers are located in the ventral region of the dorsal funiculus, showing that the corticospinal tract is labeled, though the labeling intensity differs on the right and the left sides. The dashed lines outline the corticospinal tract at the base of the dorsal funiculus. (B) Horizontal section of the upper thoracic spinal cord (intact region) of a SCI rat. Texas Red-labeled fibers extend through the corticospinal tract situated in the ventral region of the dorsal funiculus, showing that the corticospinal tract is labeled, though the labeling intensity differs on the right and the left sides. The dashed line represents the median of the spinal cord. (C) Horizontal section of the spinal cord lesion of a SCI rat. A few Texas Red-labeled axons are found (arrows) in the lesion. Scale: 200 µm for (A) and (B), and 100 µm for (C). Panels (D), (E), and (F): Immunostaining of serotonergic, monoaminergic, and CGRP-containing fibers within the lesion. Longitudinal (horizontal) section. This spinal cord was obtained from the rat that had received BMSC transplantations starting at 2 weeks after contusion injury (2-w PI), and was fixed 4 weeks following the initial cell injection. This rat showed a BBB improvement from 3 points before cell injection to 12 at fixation. (D) This panel shows serotonergic fibers (arrows). (E) This panel shows monoaminergic fibers (arrows). (F) This panel shows CGRP-containing fibers (arrows). Scale: 100 µm for (D), (E), and (F).

In tracing the axons descending from the brain, TDA-labeled corticospinal axons were clearly shown in the ventral part of the dorsal funiculus at the upper thoracic spinal cord (Figure 9A, B). A few TDA-labeled axons were found within the epicenter of the lesion (Figure 9C). This finding indicated that some axons that regrew from the corticospinal tract were included in the regenerating axonal bundles extending through the lesion of the SCI.

We also examined serotonin (5-HT)-, thyrosine hydroxylase (TH)-, and CGRP-containing axons within the lesion by immunohistochemistry. The first two are axons descending from the brain stem, while the last one belongs to the class of primary sensory axons derived from dorsal roots. These serotonergic (Figure 9D), monoaminergic (Figure 9E) and CGRP-containing axons (Figure 9F) were demonstrated to be fine fibers with chains of tiny bead-like structures. This finding indicated that both descending and ascending axons contributed to the regenerating axonal bundle extending through the epicenter of the lesion.

### Electron microscopy

**Figure 10:**
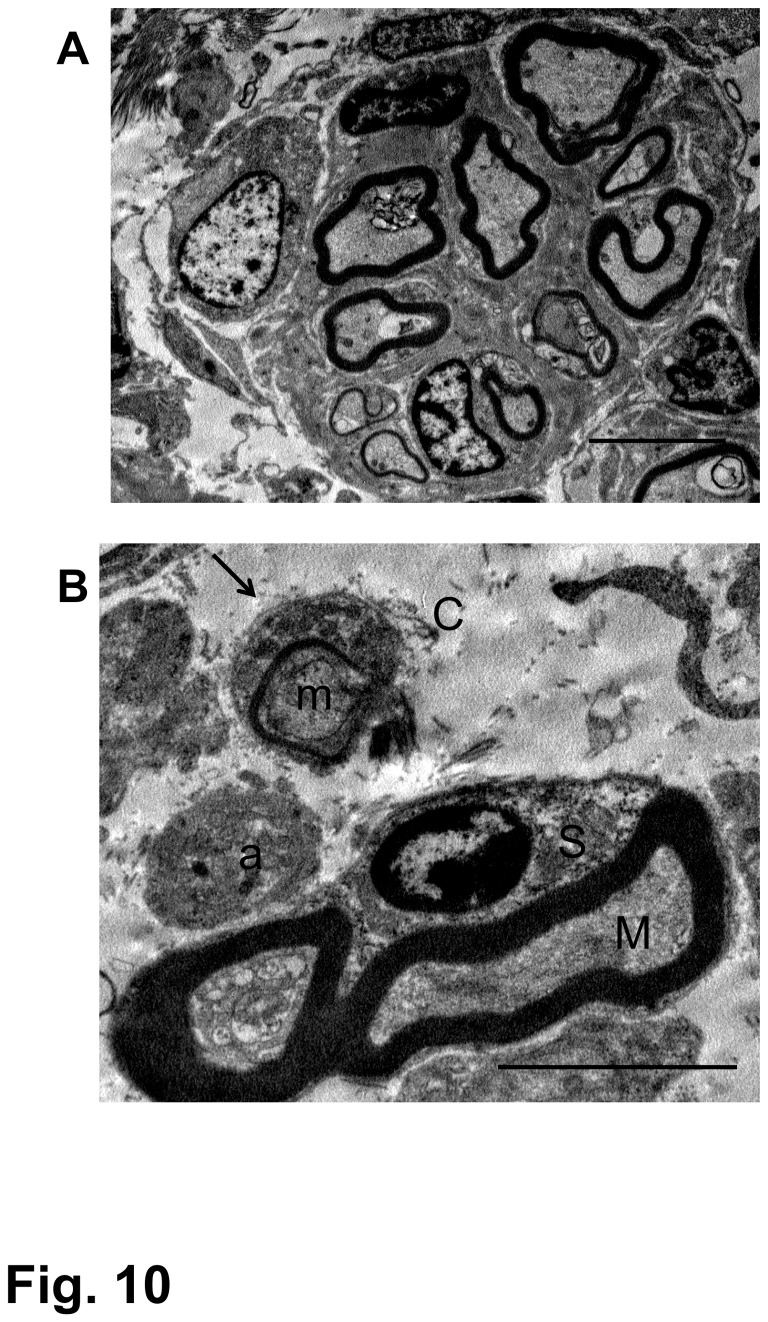
Electron microscopy of regenerating nerve fibers. These micrographs were obtained from the spinal cord epicenter of the rat that had received BMSC transplantations starting at 4 weeks after contusion injury and was fixed 4 weeks following the initial cell injection (4-w PI). This rat showed a BBB improvement from 4 points before cell injection to 10 at fixation. (A) Myelinated nerve fibers are grouped in a small fascicle, which is surrounded by a clear, cell-free space. Scale: 5 µm. (B) Part of the nerve fascicle is enlarged. There are thick-myelinated (M) and thin-myelinated (m) axons. The former is surrounded by a Schwann cell (S), and the latter is associated with a thin Schwann cell cytoplasm, the basal lamina of which is clearly seen (arrow). An unmyelinated fiber (a) is also seen near these myelinated axons. These nerve fibers are located in the space, through which collagen fibrils (C) are distributed, partly in association with Schwann cells. Scale: 5 µm.

Myelinated and unmyelinated axons in the astrocyte-devoid area were seen grouped into small fascicles (Figure 10A). This kind of axonal fascicle matched the finding of immunohistochemistry, in which axons were identified in tiny groups (Figure 8C). Electron microscopy indicated that all axons within the astrocyte-devoid area were associated with Schwann cells, and Schwann cell-associated axons were surrounded by tissues containing collagen fibrils (Figure 10B). A tissue with extracellular matrices containing collagen fibrils can be regarded as a connective tissue. Axons associated with Schwann cells in such connective tissue matrices (CTM) are of a peripheral nature. These findings were the same in all three groups. Axons varied in diameter, ranging from less than 1 µm without myelin sheath to over 4-5 µm with myelin sheath. The fact that axons had varying degrees of diameter and were located in the newly produced CTM indicates that they were not original spinal cord axons that had survived after demyelination due to injury, but were regenerated axons that had grown out in the CTM. Attention was paid to ensure that electron microscopic examination should be directed to axons located within astrocyte-devoid CTM areas. Axons that were located within the astrocyte-present area, or were apparently continuous with dorsal roots, were excluded from the observation. Astrocytes and oligodendrocytes did not invade the epicenter of the CTM-occupied lesion, indicating that glial cells could not contribute to the axonal outgrowth within the lesion. The CTM might have served as a scaffold for the growth of regenerating axons.

### Fate of BMSCs

**Figure 11:**
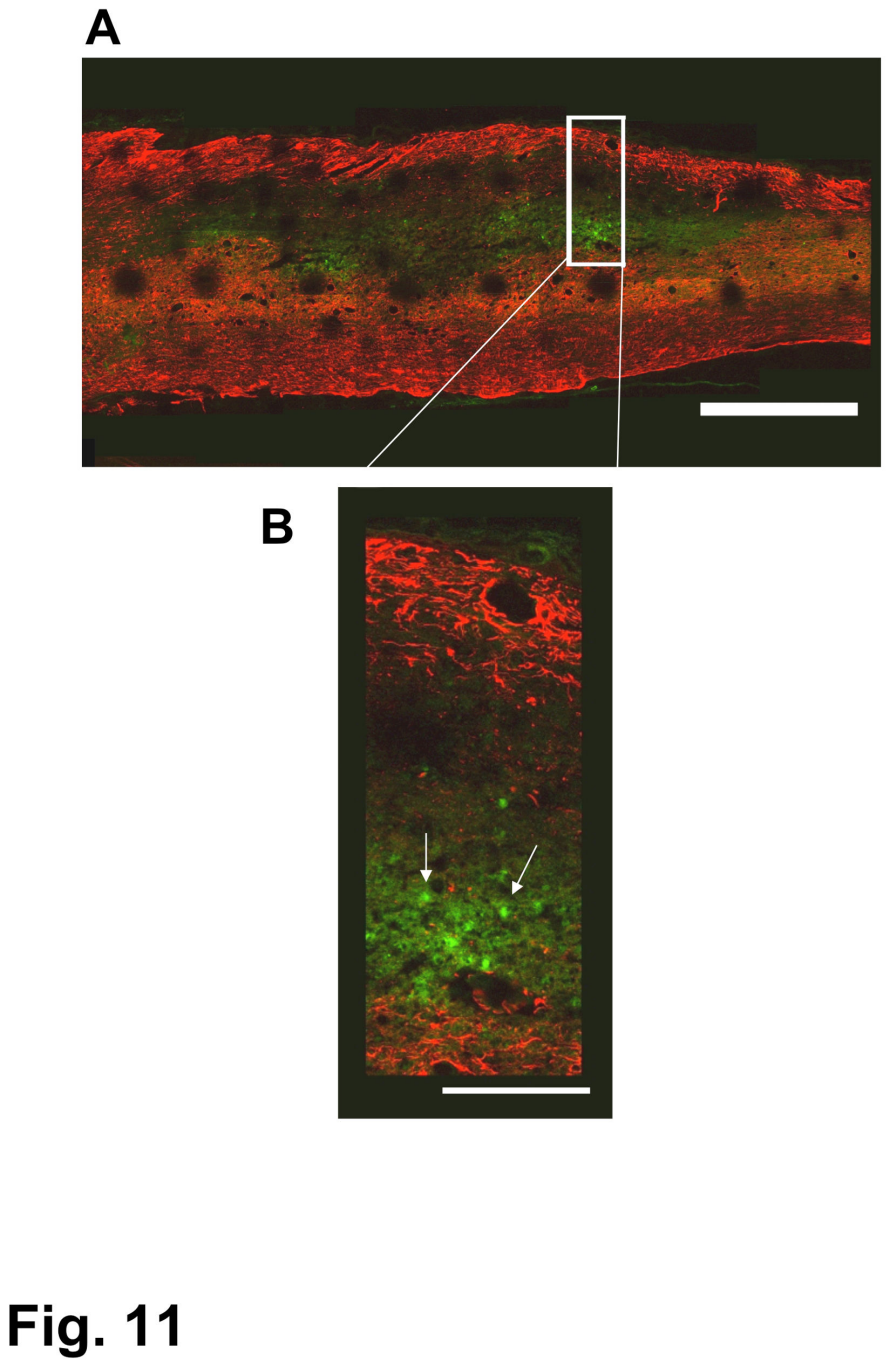
BMSCs in the spinal cord lesion of the 1-w PI group. A: A few BMSCs (green) are found within the lesion 2 days after transplantation. B: The region of the rectangle was enlarged to show BMSCs (arrows) within the lesion. Astrocytes are immunostained red. Scale: 1 mm for (A), 250 µm for (B).

A small number of green-fluorescent GFP-transgenic BMSCs were found within the spinal cord lesion at 2 days, but none at 7 days post-initial transplantation in the SCI of the 1-w PI group (Figure 11). At 2 or 7 days after transplantation, no BMSCs were found in the spinal cord lesion in the SCI of 2-week and 4-week PI groups. We also examined preparations selected from rats fixed at 2 and 4 weeks after initial cell transplantation. There were no GFP-transgenic BMSCs in the spinal cord lesion or on the adjacent spinal cord surface in those preparations.

### CSF effects on neuronal survival and neurite extension

**Figure 12:**
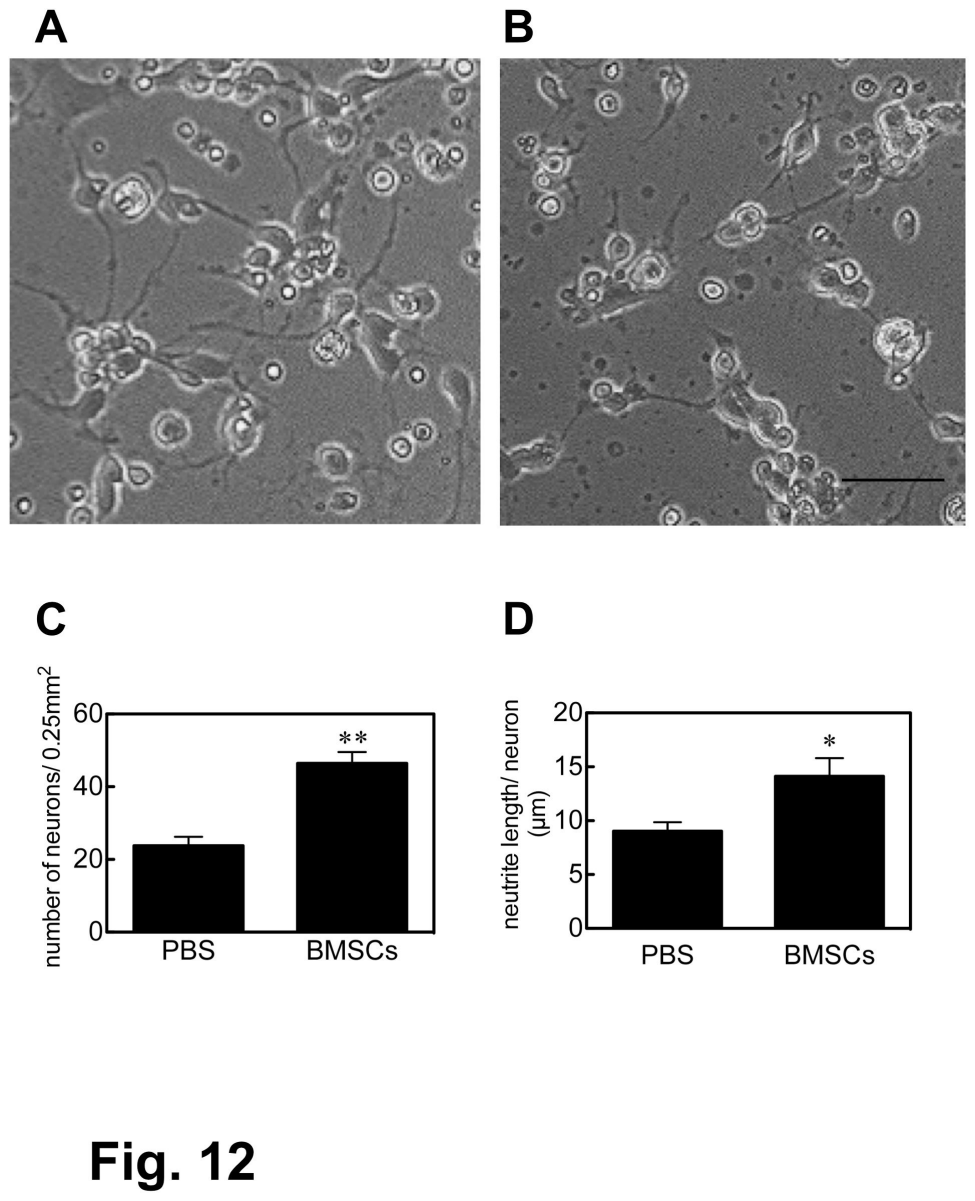
The CSF effect on cultured hippocampus neurons. The effects of BMSC-transplanted (A) and PBS-injected (B) CSF on the survival and the neurite extension of cultured hippocampus neurons were examined. Neuronal survival and neurite extension were quantified in (C) and (D), respectively (* P<0.05, ** P<0.01).. Scale: 100 µm.

Micrographs of hippocampus neurons cultured in the medium containing CSF are shown in Figure 12A and 12B. Two days after transplantation, the density of neurons was 46.4 ± 3.5 and 23.0 ± 2.7/0.25 mm^2^ (ｐ < 0.01) in BMSC- and PBS-delivered CSF, respectively, and the neurite length per neuron was 14.1 ± 2.3 and 9.0 ± 1.5 µm/neuron (ｐ < 0.05) in BMSC- and PBS-delivered CSF, respectively, (Figure 12C, D). Our observations indicated that there was no significant difference between BMSC- and PBS-delivered CSF at 7 days of transplantation.

### Locomotor recovery

**Figure 13:**
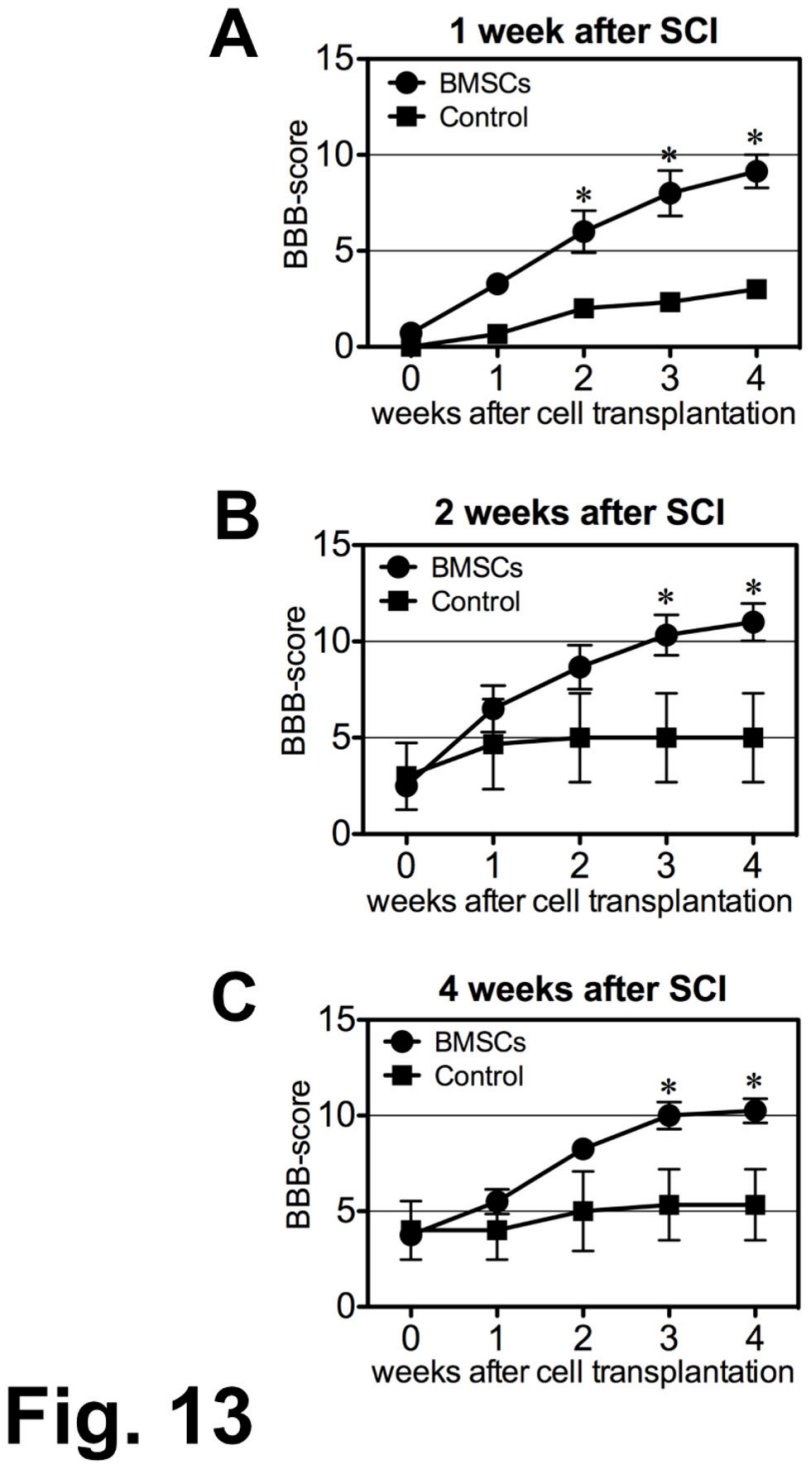
Locomotor evaluation. BMSCs were injected into the CSF three times (once weekly) at 0, 1 and 2 weeks beginning at 1-w, 2-w, and 4-w PI as indicated on the abscissa in the graph. The rats were observed for 4 weeks following the initial injection. (A) The BMSC transplantation was performed at 1-w PI. The locomotor improvement becomes obvious from 2 weeks post-initial injection (pIn). The average BBB scores reach 9.0 ±1.0 points (mean ± SEM) at 4 weeks pIn. The average BBB score of the control rats remains at 3.0 ± 0.5 points (*: p < 0.01).. (B) The BMSC transplantation was performed at 2-w PI. The locomotor improvement becomes obvious from 3 weeks pIn. The average BBB scores are 10.9 ± 2.2 points at 4 weeks pIn. The BBB score of the control rats remains at 5.0 ± 2.1 points (*: p < 0.05 at 3weeks, and p < 0.01 at 4 weeks pIn).. (C) The BMSC transplantation was performed at 4-w PI. The locomotor improvement is gradual. The average BBB scores reach 10.2 ± 1.0 points at 4 weeks pIn. The BBB score of the control rats remains at 5.1 ± 1.7 points. (*: p < 0.01 at 3 and 4 weeks pIn.)

Rats showed low BBB scores (less than 7 points) before cell or vehicle injection. The average BBB scores differed somewhat in the three groups at the time of cell or vehicle injection. There was a prominent elevation in the BBB scores of cell-transplanted rats in all groups, whereas BBB scores were only slightly changed in the vehicle-injected control rats (Figure 13). In the 1-w PI group, BMSC-transplanted rats showed a marked BBB elevation from 1.0 ± 0.4 points before injection to 9.0 ± 1.0 points at 4 weeks post-initial injection (pIn), while the control rats showed 0.5 ± 0.3 points before injection, and 3.0 ± 0.5 points at 4 weeks pIn. There was a significant difference (ｐ < 0.01) between the BMSC-transplanted and the control rats at 2-4 weeks pIn (Figure 13A). In the 2-w PI group, the BMSC-transplanted rats showed an elevation from 3.0 ± 1.5 points before injection to 10.9 ± 2.2 points at 4 weeks pIn, while the control rats showed 3.5 ± 1.5 points before injection, and 5.0 ± 2.1 at 4 weeks pIn. The difference in BBB scores was significant at 3 weeks (ｐ < 0.05) and 4 weeks (ｐ < 0.01) pIn (Figure 13B). In the 4-w PI group, BMSC-transplanted rats showed a distinct elevation from 3.5 ± 1.5 points before injection to 10.2 ± 1.0 points at 4 weeks pIn, while the control rats showed 4.0 ± 1.5 points before injection and 5.1 ± 1.7 points at 4 weeks pIn. There was a significant difference (ｐ < 0.01) between BMSC-transplanted and control rats at 3 and 4 weeks pIn (Figure 13C).

## Discussion

The present study showed that the infusion of BMSCs through the CSF has beneficial effects on locomotor recovery and tissue repair, including axonal outgrowth, in the subacute (1-w and 2-w PI) and chronic (4-w PI) SCI of rats.

### Tissue repair

There is no sign suggesting a migration of surviving glial cells from the surroundings into the lesion. It seems that the tissue repair was due mainly to the presence of non-neural tissues, including extracellular matrices composed of collagen fibrils. The source of cells involved in the production of non-neural tissues or CTM is not identified in the present study. However, it is probable that the CTM cells came partly from meningeal and partly from periarterial regions. Non-neural cells accompanying large vessels are found in the perivascular spaces in the CNS [23].

The glial scar, including connective tissue formation, has been regarded as an impediment to axonal regeneration [24]. Chondroitinase ABC treatment reduces the inhibiting activity of a glial scar, leading to axonal regeneration [25]. An extensive glial scar is usually formed in an open incision injury such as a transection or hemisection to protect the CNS tissue from the invasion of extrinsic non-neural tissues (24). It was not determined in the present study whether proteoglycans accumulated at the border of the lesion. As far as astrocytic gliosis is concerned, however, no distinct glial scar was formed at the border of the lesion in the present contusion injury study. The present study indicates that the CTM formed in the spinal cord might not be an impediment, but might be, rather, supportive as the scaffold for the growth of regenerating axons. This finding is the same as that in our previous study, in which BMSCs were transplanted directly into the subacute contusion injury of the spinal cord [10]. It can be said that a contusion spinal cord injury provides more plausible conditions for axonal regeneration than an incision injury. Clinically, a contusion injury might be more common than an incision injury, indicating that a contusion injury effected in research would approximate more closely the typical clinical spinal cord injury.

In terms of tissue repair in the spinal cord lesion, endogenous progenitor or stem cell proliferation should be taken into consideration. Endogenous progenitor cells have been demonstrated to proliferate in response to an injury in the spinal cord [26,27]. Ependymal cells of the central canal are considered to be one of the main sources of progenitor or stem cells in the SCI [28,29]. Although there is a finding suggesting the proliferation of ependymal cells in rat SCI, it is not clear to what extend ependymal cell-derived progenitor or stem cells contribute to the tissue repair and axonal outgrowth in an injured spinal cord.

### Axonal outgrowth

Numerous axons extended through the CTM within the lesion. There is a possibility that some host axons survived after demyelination in the injured sites. Such demyelinated axons usually show almost uniform diameter and smooth contour throughout their length. Electron microscopy in the present study showed that axons in astrocyte-devoid areas had varying degrees of diameter with rough contours, suggesting that axons within astrocyte-devoid areas might be newly extended ones.

The CTM appear to serve as effective scaffolds for the outgrowth of regenerating axons. It should be noted that axons are associated with Schwann cells. This is the pattern of peripheral nerve regeneration. In this study, the source of Schwann cells was not determined. It is conceivable that Schwann cells associated with root fibers came into contact with the injured spinal cord tissue, through which they migrated into the spinal cord and were in contact with growing axons. It is probable that growth of the axons was promoted by Schwann cells.

No glial cell migration was found in the CTM, implying that glial cells were not associated with the axonal outgrowth within the CTM. The same distinct nerve regeneration through the CTM was also found in our previous study [10]. It is generally believed that the extracellular matrices composed of proteoglycans, collagen fibrils, and fibronectin are inhibitory to the growth of regenerating axons in the central nervous system [30,31,32]. On the other hand, however, it is reported that some proteoglycans, collagen type IV and laminin are associated with the growing axons in the SCI [33]. Similarly, decorin [34], hyaluronic oligosaccharides [35] and tenascin-C [36] are reported to promote axonal outgrowth in SCI. Fibroblasts co-transplanted with neural progenitor cells enhance axonal regeneration in the SCI [37]. In vitro, BMSCs stimulate axonal outgrowth over inhibitory molecules, including proteoglycans and myelin-associated glycoproteins [38]. These studies suggest that axonal outgrowth might have been supported by the CTM formed in the lesion in the present study.

In the present study, numerous regenerating axons extended through the lesion. There was no finding suggesting a blocked extension of regenerating axons at the rostral or caudal borders. It appeared that axons traversing the lesion extended into the rostral and caudal borders. It is shown that these axons include corticospinal, raphespinal, and coerulospinal fibers, indicating that axons descending from the brain or brain stem grew out into the lesion. The presence of CGRP-containing axons indicates that ascending fibers also contribute to axonal regeneration. An apparent extension of regenerating axons into the rostral and caudal borders suggests that these regenerating axons might form neural connections with host neural networks. This may be the cytological evidence for the prominent locomotor improvement in the present study.

SCI has been studied extensively at its acute phase [1,2]. However, the subacute and chronic SCI are clinically more important [6,7,19,20,21,39]. It is reported that the chronic SCI might have lost its plasticity to respond to trophic factors [11]. However, the present study demonstrated that BMSCs promoted axonal outgrowth in the chronic SCI (4-w PI). This indicates that the plasticity for nerve regeneration can persist at least 4 weeks after a contusion injury in rats. The direct injection of BMSCs into the lesion is not needed, but the infusion of BMSCs through the CSF is effective. This is a basis for clinical application of BMSCs by lumbar puncture for patients with subacute or chronic SCI.

### The role of BMSCs

The space of the central canal of the spinal cord is so limited that BMSCs injected into the fourth ventricle might not flow through the central canal. BMSCs go from the fourth ventricle via the foramina of Luschka and Magendie into the subarachnoidal space including the cisterna magna, and flow caudally through the subarachnoidal space of the spinal cord. In this process, some BMSCs migrated into the lesion from the injured spinal cord surface. The present study showed that a small number of BMSCs entered the spinal cord lesion and survived there for only a short time (2-7 days) after transplantation in the 1-w PI group. However, there was no finding suggesting that BMSCs entered the spinal cord lesion in the rats of the 2-w and the 4-w PI groups. It appeared, in fact, that BMSCs could not migrate into the lesion in the 2-w and 4-w PI groups, possibly indicating that the spinal cord lesion was “sealed”, no longer penetrable by BMSCs.

BMSCs, even when having entered the lesion, did not survive therein long enough to be integrated into the host spinal cord tissue. This finding is in keeping with our previous studies [3,10]. BMSCs, therefore, did not serve as the scaffolds for the tissue repair that included axonal outgrowth in SCI.

Although BMSCs soon disappeared after transplantation, an extensive axonal outgrowth occurred in the spinal cord lesion in the three groups. This leads to the hypothesis that BMSCs released certain trophic factors that might work to promote tissue repair, including axonal regeneration [3,15,21,39,40,41]. Our previous studies demonstrated that BMSC-delivered CSF had neurotrophic effects on neurospheres in vitro [3], and that the concentration of HGF was elevated in the CSF into which bone marrow-derived mononuclear cells had been injected [15]. The preliminary experiment in the present study showed that the BMSC-delivered CSF promoted the survival and neurite extension of cultured hippocampus neurons, as we had also found in our previous study [3]. It can be said that BMSCs have an effect through the CSF by releasing certain neurotrophic factors effective for the survival and neurite extension of neurons. And, indeed, the injection of BMSCs contributed to the reduction in volume of the spinal cord cavity, and to the promotion of functional recovery in SCI mice, presumably through the neurotrophic functions of BMSCs [42]. There was no finding suggesting the differentiation of BMSCs into neurons or glial cells that include astrocytes and oligodendrocytes [42].

In fact, there are many reports that BMSCs secrete various kinds of trophic factors including VEGF, IGF, HGF, bFGF, and GDNF [15,19,22,43,44,45], and certain extracellular matrix molecules such as laminin and type IV collagen [46]. BMSCs also regulate the caspase-3 mediated apoptosis pathway, indicating that axons are protected from degenerative changes following SCI [47]. Our previous in vitro study demonstrated that BMSCs secrete a variety of trophic factors for neuronal survival and neurite extension, and that no single trophic factor is as efficient as the conditioned medium of BMSC culture [22]. This suggests that combinatorial effects of trophic factors might contribute mainly to spinal cord tissue repair, including axonal outgrowth, rescue of degenerating axons, and proliferation of the CTM.

BMSCs were injected 3 times in the present study. Three intrathecal catheter deliveries (once daily) of BMSCs yield a higher behavioral improvement than a single injection in the rat SCI [48]. Although the data comparing the efficacy of single and triple injections (once weekly) in the subacute or chronic SCI are not available in the present study, it is reasonable to suppose that the repetitive three-time infusion is more effective than the single infusion. However, this problem is not critical from the clinical point of view, since multiple injections of BMSCs should be less of a burden on patients and can be readily performed by lumbar puncture in the clinical application [17,18].

### Locomotor improvement

The BBB scores showed only a small elevation in the control subgroups, whereas a marked elevation was noted in BMSC-transplanted subgroups. The locomotor improvement is clearly manifested in both subacute and chronic SCI. This prominent recovery of behavior might be attributed to the numerous regenerating axons and their smooth transition into the host neural tissues at the rostral and caudal borders. This suggests that descending and ascending fibers might connect with the host neural networks, contributing to locomotor improvement. Regeneration of corticospinal axons plays an important role in functional recovery in rats [49], and the correlation between locomotor recovery and nerve regeneration in SCI rats has been demonstrated in many other studies [50,51]. The extensive axonal regeneration implies the efficacy of rehabilitation exercise [52,53]. The mechanism for this efficacy is an interesting problem in the SCI. Future animal experiments are needed to reveal the relationship between neural connectivity development, including synapse formation, and behavioral improvement.

### Clinical perspectives

A “regeneration” study with any significance must be applicable to clinical uses. The present study indicates that BMSC infusion through the CSF is effective even in the chronic SCI in rats. This means that patients who are suffering from SCI might be treatable by BMSC infusion by lumbar puncture. BMSCs are promising transplants: they have a very noticeable effect and are safe in that they can be used as autologous transplants. The lumbar puncture is widely used clinically. Therefore, BMSC transplantation by lumbar puncture is clinically a suitable method. Several clinical BMSC transplantations have been reported [54,55,56]. We have been carrying out a I/II study of BMSC transplantation in patients with SCI since 2007 [17,18]. In that study, BMSCs were obtained from an acute SCI patient, expanded by culture for 10-15 days, and transplanted to him by lumbar puncture. The patients, therefore, were in the subacute condition at the time of cell transplantation. The present study has demonstrated that BMSC infusion into the CSF improves chronic as well as subacute SCI. This is important in that it indicates the feasibility of applying BMSC transplantation by lumbar puncture to patients currently suffering from SCI.
